# Deterioration of COVID-19 vaccine efficacy by antipsychotic medications in schizophrenia patients

**DOI:** 10.1128/spectrum.00021-25

**Published:** 2025-03-31

**Authors:** Takashi Kusunoki, Naomi Tsubata, Nariaki Iijima, Shiho Mitsugi, Toru Nagasaka, Takeshi Senga, Ryoichi Ichie, Takashi Okamoto

**Affiliations:** 1 Junwakai Yahagigawa Hospital, Anjo, Aichi, Japan; 2 Departments of Public Health, Nagoya City University Graduate School of Medical Sciences, Mizuho-ku, Nagoya, USA; 3 Departments of Molecular and Cellular Biology, Nagoya City University Graduate School of Medical Sciences, Mizuho-ku, Nagoya, Japan; Kumamoto Daigaku, Kumamoto, Kumamoto, Japan

**Keywords:** schizophrenia, antipsychotics, risperidone, aripiprazole, olanzapine, COVID-19 vaccination, vaccine efficacy

## Abstract

It has been previously documented that some antipsychotic medications could deteriorate the protective efficacy of vaccines. Likewise, we have initially observed that a significantly increased number of schizophrenic inpatients became ill irrespective of COVID-19 vaccination compared to healthy hospital workers. Thus, we examined the effects of anti-psychiatric medication on the COVID-19 incidence. A cohort study was conducted with 98 schizophrenia inpatients and 78 control hospital staff members, all of whom received the COVID-19 vaccine between July and September 2021. COVID-19 incidence and vaccine-related side effects were compared between these two groups. The schizophrenia group had a significantly higher incidence of COVID-19 (40.8%) compared to the control group (12.8%) (*P* < 0.01). In particular, risperidone monotherapy was associated with a significant increase in COVID-19 incidence (65.6%), whereas aripiprazole use was linked to a significant reduction (0%) (*P* < 0.01). No such difference in COVID-19 incidence was observed in patients using olanzapine. Risperidone administration appears to impair the immune responses and reduce vaccine effectiveness. Further studies with extended subjects and other vaccines are warranted to clarify the anti-vaccine effects associated with risperidone.

**IMPORTANCE:**

The vaccine efficacy in this study is significant because it sheds light on the potential impact of antipsychotic medications, particularly risperidone, on the effectiveness of COVID-19 vaccines in individuals with schizophrenia. The findings suggest that patients treated with risperidone have a significantly higher risk of incidence of COVID-19 despite vaccination, highlighting a possible interference with immune responses. In contrast, aripiprazole appears to have a protective effect, and the combined use of both medications results in an alarming increase in COVID-19 incidence. These results underscore the importance of further investigating the immunological mechanisms underlying these effects, especially through the dopamine receptor activity. Understanding these interactions could lead to more effective vaccination strategies and better healthcare management for patients with schizophrenia, ensuring better protection against COVID-19 and other infectious diseases.

## INTRODUCTION

The global outbreak of COVID-19, caused by the coronavirus known as SARS-CoV-2 (1, 2), was marked by its high infectivity and severe symptoms. The World Health Organization (WHO) designated it as a Public Health Emergency of International Concern (PHEIC). As of October 2024, over 770 million infections and 7.07 million deaths have been reported globally (https://covid19.who.int/). The risks of complications and mortality due to COVID-19 are particularly high among patients in long-term care facilities (3) and increase with factors such as age, the presence of metabolic syndrome, or diabetes (4, 5). Among these, psychiatric inpatients with severe mental disorders have been reported to exhibit heightened vulnerability to COVID-19 (6, 7), especially those with schizophrenia, characterized by limited physical activity and prolonged use of potent antipsychotics (8).

Concerns have been raised regarding unnoticed pharmacological effects associated with antipsychotics (9), which frequently induce side effects such as hypertension, dyslipidemia, and diabetes, all of which are known to increase the risk of COVID-19 (10–12). Consequently, many countries have prioritized vaccination for such individuals. Furthermore, certain antipsychotics have been reported to interfere with immune responses (13–15). In this study, we compared the incidence of COVID-19 among schizophrenia patients vaccinated against COVID-19 while receiving various antipsychotic medications and raised a concern that the vaccine’s efficacy may be substantially diminished in individuals treated with certain drugs.

## MATERIALS AND METHODS

The patient group comprised 98 inpatients with schizophrenia admitted to our psychiatric ward between April 2021 and August 2024, 93 of whom were on various antipsychotics. Most patients were given risperidone, aripiprazole, or olanzapine. There were no significant differences in the clinical subtype of schizophrenia or its severity between those treated with risperidone and those who were not (data not shown). The control group included 78 hospital staff members. All participants received the COVID-19 vaccine (0.3 mL COMIRNATY, Pfizer, administered intramuscularly) between July and September 2021. The patient group included 49 men and 49 women, whereas the control group had a significantly higher proportion of women (17 men and 61 women, *P* < 0.01). There were no notable differences in ages: 17 patients and 15 controls were under 40, while 82 patients and 63 controls were 40 years or older. Adverse reactions (e.g., injection site pain, fatigue, or fever ≥38.0°C) were recorded after the first and second doses. The incidence of COVID-19 over one year post-vaccination was evaluated in both groups. Fisher’s exact test was used for statistical analyses, with *P* < 0.05 considered significant. Control data for vaccine adverse reactions were obtained from the Phase I/II trial (C4591005 study) in Japan in 2021 (https://labeling.pfizer.com/ShowLabeling.aspx?id=15700).

## RESULTS

Upon COVID-19 vaccination, a significant difference in the frequency of side effects was observed. As shown in Table 1, the patient group experienced side effects at a rate of 46.9%, which was notably lower than that of the control (92.3%) (*P* < 0.01). The incidence of side effects following the first and second doses of the vaccine in the patient group was as follows: pain at the injection site (14.8% and 18.2%, *P* < 0.01), fatigue (3.4% and 5.1%, *P* < 0.01), and fever above 38°C (1.1% and 5.1%, *P* < 0.05), all of which were less frequent compared to the control group. Although 92.3% of healthy control subjects suffered from any side effects throughout the first and second COVID-19 vaccination, much less (46.9%) patients experienced the vaccine side effects (Table 2). More importantly, a significant difference in COVID-19 incidence was also observed between the two groups (Table 3). The patient group had a COVID-19 incidence rate of 40.8%, whereas the control group had an incidence of 12.8% (*P* < 0.01). This observation indicates a significantly higher rate of COVID-19 occurrence in schizophrenia patients.

**TABLE 1:** Vaccine-associated adverse reactions in patients and control subjects

Items	First vaccination (*n* = 176)			Second vaccination (*n* = 176)		
	Patient (*n* = 98)	Control (*n* = 78)	*P*-value	Patient (*n* = 98)	Control (*n* = 78)	*P*-value
Pain, *n* (%)	Yes 26 (14.8)	63 (35.8)	<0.001	32 (18.2)	58 (33.0)	<0.001
	No 72 (40.9)	15 (8.5)		66 (37.5)	20 (11.4)	
Fatigue, *n* (%)	Yes 6 (3.4)	29 (16.5)	<0.001	9 (5.1)	48 (27.3)	<0.001
	No 92 (52.3)	49 (27.8)		89 (50.6)	30 (17.0)	
Fever, *n* (%)	Yes 2 (1.1)	8 (4.8)	<0.05	9 (5.1)	36 (20.5)	<0.001
(>38°C)	No 96 (54.5)	70 (39.8)		89 (50.6)	42 (23.9)	

**TABLE 2:** Adverse reactions that occurred in one or more doses of the first or second vaccination

Items	Patient (*n* = 98)	Control (*n* = 78)	*P*-value
Adverse reactions (yes)	46 (46.9%)	72 (92.3%)	<0.001
Adverse reactions (no)	52 (53.1%)	6 (7.7%)	

**TABLE 3:** Comparison of infection status one year after COVID-19 vaccination

Items	Patient (*n* = 98)	Control (*n* = 78)	*P*-value
COVID-19 infection (yes)	40 (40.8%)	10 (12.8%)	<0.001
COVID-19 infection (no)	58 (59.2%)	68 (87.2%)	

We then analyzed the incidence of COVID-19 among schizophrenia patients based on antipsychotic medication administered. Risperidone, aripiprazole, and olanzapine were the most frequently prescribed medications in the patients, often in combination. There was no significant difference in the frequency of side effects among the different antipsychotic medications (data not shown). However, a notable difference in the COVID-19 incidence was observed depending on the medication administered. As shown in Table 4, COVID-19 incidence was 65.6% in patients receiving risperidone alone, compared to 6.2% in those not receiving risperidone, showing more than a tenfold increase in the disease incidence (*P* < 0.01). In contrast, the incidence of COVID-19 in patients receiving aripiprazole alone was 0%, while those not receiving it had an incidence of 42.5%, suggesting a significant protective effect of aripiprazole (*P* < 0.01). In those patients receiving both risperidone and aripiprazole (only six cases), all six developed COVID-19 (100% incidence), although the number of such patients was very low (*P* < 0.01). Among the 36 patients who did not take either medication, only 3 (8.3%) developed COVID-19 (Table 4). These findings suggest that both risperidone and aripiprazole appear to interfere with the immune response to the COVID-19 vaccine but in opposite directions. No significant difference in COVID-19 incidence was observed between patients receiving olanzapine alone (25.0%) and those not receiving it (39.7%).

**TABLE 4:** Effects of anti-psychiatric medication on the COVID-19 incidence

Items	*n* (%)		Occurrence of COVID-19 (+)	Occurrence of COVID-19 (−)	*P*-value
Risperidone	*n* = 48	None	3 (6.2%)	45 (93.7%)	<0.001
	*n* = 32	Monotherapy	21 (65.6%)	11 (34.3%)	<0.001
Aripiprazole	*n* = 80	None	34 (42.5%)	46 (57.5%)	<0.001
	*n* = 12	Monotherapy	0 (0%)	12 (100%)	<0.001
Olanzapine	*n* = 68	None	27 (39.7%)	41 (60.2%)	ns
	*n* = 12	Monotherapy	3 (25.0%)	9 (75.0%)	ns
Risperidone + Aripiprazole	*n* = 6	Yes	6 (100%)	0 (0%)	<0.001
	*n* = 36	No	3 (8.3%)	33 (91.7%)	<0.001
Risperidone + Olanzapine	*n* = 12	Yes	10 (83.3%)	2 (16.7%)	<0.001
	*n* = 30	No	0 (0%)	30 (100%)	<0.001

Additionally, another COVID-19 outbreak occurred in one of the previously affected wards two years later. This time, the outbreak involved 24 patients, all of whom had received the COVID-19 vaccine. While the similar effect of risperidone use on the COVID-19 incidence was still noted, the difference was not statistically significant (Table 5).

**TABLE 5:** Two-year follow-up of vaccinated patients with risperidone therapy*^a^*^,^*^b^*

Initial phase (*n* = 31)				Second phase (*n* = 24)		
Patient group	Risperidone administration		*P*-value	Risperidone administration		*P*-value
	Present	Absent		Present	Absent	
SARS-CoV-2+, *n* (%)	17 (100)	0 (0)	<0.001	7 (100)	0 (0)	ns
SARS-CoV-2−, *n* (%)	5 (35.7)	9 (64.3)		11 (64.7)	6 (35.3)	

^*a*^ The 1st COVID19 out brake was Jul 2022, wheres the 2nd outbreak was Aug 2024.

^*b*^ Fisher's exact probability test for categorical variables and Wilcoxon rank sum test for continuous variables.

## DISCUSSION

It is known that numerous psychiatric medications often interfere with immune responses (13–15), thus raising a concern about their potential to impair the efficacy of COVID-19 vaccination (16). To investigate the efficacy of COVID-19 vaccines in preventing SARS-CoV-2 infection among schizophrenia patients, we conducted a study involving 98 patients from our hospital. This investigation was prompted by an outbreak within the facility, allowing us to collect detailed longitudinal data on patients’ clinical histories and medication regimens. All participants were inpatients, enabling detailed follow-up and analysis of the relationship between COVID-19 onset and vaccine efficacy.

We found significant differences between the schizophrenia patient group and the control group in the incidence of COVID-19 as well as the vaccine-related adverse reactions. Notably, the type of antipsychotic medication administered significantly influenced the vaccine’s effectiveness. Among schizophrenia patients, those receiving risperidone monotherapy exhibited a significantly higher incidence of COVID-19 (*P* < 0.01). Conversely, those on aripiprazole monotherapy showed a significant reduction in incidence (*P* < 0.01). For olanzapine monotherapy—the second most frequently prescribed antipsychotic after risperidone in this hospital—there was no significant difference in the COVID-19 incidence. When risperidone was co-administered, both aripiprazole and olanzapine were associated with an increased incidence of COVID-19 (*P* < 0.01). For those patients under the treatment of both risperidone and aripiprazole, the vaccine’s preventative effect was almost entirely nullified. These findings were independent of schizophrenia subtype, age, sex, or the use of non-antipsychotic medications (e.g., antihypertensives or lipid-lowering agents). No differences were observed concerning dosage variations in antipsychotics.

The study had certain limitations: (i) a relatively small sample size, (ii) the inability to measure antibody titers or cellular immune responses in the study subjects, and (iii) the limited ethnicity of all Japanese individuals. Thus, further studies with expanded subjects and further immunological examinations, such as the detection of anti-SARS CoV2 antibodies and the measurement of antiviral T-cell responses, should give more definitive conclusions.

Our findings suggest that antipsychotics could interfere with immune responses, warranting further exploration of their underlying mechanisms and pharmacological effects. Although studies on the impact of COVID-19 vaccines on schizophrenia patients remain limited, accumulating evidence supports the relevance of this topic. For example, Nemani et al. (17) recently reported that schizophrenia is associated with significantly reduced levels of SARS-CoV-2 spike protein antibodies; however, their study did not account for antipsychotic use. In addition, O’Brien et al. (18) documented reduced vaccine efficacy among schizophrenia patients treated with clozapine. Since risperidone and clozapine share α₁-receptor agonist activity, a property absent in aripiprazole and olanzapine (19), our observations are relevant to their notion. Given the role of α₁-receptors in early immune responses (20–23), their involvement could explain the interference with vaccine efficacy observed in our study. Additionally, risperidone and clozapine have been shown to reduce TNF-α levels and increase IL-10 concentrations (24, 25), which usually occur at the initial phase of immune responses, further supporting our findings.

### Conclusions

The phenomena we have reported here are not entirely unforeseen. Thus, the impact of antipsychotic medications on the efficacy of COVID-19 vaccines, as well as their broader implications for the prevention and management of infectious diseases, has highlighted an important area for future investigation. We hope our findings can facilitate the experimental approaches to elucidate the effects of antipsychotics on various stages of immune responses. Moreover, the influence of antipsychotic medications on vaccine efficacy is unlikely to be limited to COVID-19 vaccines. These findings may help to develop immune-modulating compounds based on the chemical structures of risperidone or aripiprazole.

## Data Availability

The data obtained will be available in an anonymized format so that each individual should not be identified. Data requests must be made to the author.
